# Association Between Response to Nivolumab Treatment and Peripheral Blood Lymphocyte Subsets in Patients With Non-small Cell Lung Cancer

**DOI:** 10.3389/fimmu.2020.00125

**Published:** 2020-02-07

**Authors:** Selene Ottonello, Carlo Genova, Irene Cossu, Vincenzo Fontana, Erika Rijavec, Giovanni Rossi, Federica Biello, Maria Giovanna Dal Bello, Marco Tagliamento, Angela Alama, Simona Coco, Simona Boccardo, Irene Vanni, Guido Ferlazzo, Lorenzo Moretta, Francesco Grossi, Maria Cristina Mingari, Paolo Carrega, Gabriella Pietra

**Affiliations:** ^1^Department of Experimental Medicine (DiMES) and Center of Excellence for Biomedical Research (CEBR), University of Genoa, Genoa, Italy; ^2^Lung Cancer Unit, IRCCS Ospedale Policlinico San Martino, Genoa, Italy; ^3^Center for Autoinflammatory Diseases and Immunodeficiencies - Pediatric Clinic and Rheumatology, Giannina Gaslini Institute, Genoa, Italy; ^4^Clinical Epidemiology Unit, IRCCS Ospedale Policlinico San Martino, Genoa, Italy; ^5^Medical Oncology Unit, Fondazione IRCCS Ca' Granda Ospedale Maggiore Policlinico, Milan, Italy; ^6^Department of Medical, Surgical and Experimental Sciences, University of Sassari, Sassari, Italy; ^7^Laboratory of Immunology and Biotherapy, Department of Human Pathology, University of Messina, Messina, Italy; ^8^Division of Clinical Pathology, University Hospital Policlinico G. Martino, Messina, Italy; ^9^Cell Factory Center, University of Messina, Messina, Italy; ^10^Department of Immunology, IRCCS Bambino Gesù Children's Hospital, Rome, Italy; ^11^Immunology Unit, IRCCS Ospedale Policlinico San Martino, Genoa, Italy

**Keywords:** nivolumab, PD-1, biomarkers, non-small-cell lung cancer, peripheral blood, immune checkpoint

## Abstract

Immune checkpoint blockade represents a major breakthrough in advanced non-small cell lung cancer (NSCLC) therapy. However, success is limited to a subset of patients and there is a critical need to identify robust biomarkers associated with clinical response. In this study, we assessed whether pre-existing immunological characteristics, as well as immune parameters measured during treatment, might provide such clinical guidance. We studied blood samples collected at baseline and during treatment in a cohort of advanced NSCLC patients (*n* = 74) treated with nivolumab. Several lymphocyte subsets and biomarkers were then correlated with overall survival (OS) as well as clinical response, assessed using RECIST criteria. We found that patients characterized by longer OS had higher levels of CD3^+^, CD4^+^, and CD8^+^ T cells but lower levels of NK cells at baseline. Moreover, that they displayed a statistically significant lower expression of PD-1 on both CD3^+^ and CD8^+^ T cells (*p* = 0.013 and *p* = 0.033, respectively). The pre-treatment level of exhausted T cells (CD8^+^PD1^+^Eomes^+^) was significantly lower in patients with controlled disease (CD), defined as partial response (PR), and stable disease (SD), compared to those with progressive disease (PD) (*p* = 0.046). In CD patients, the frequency of exhausted CD8^+^ T cells further decreased during treatment cycles (*p* = <0.0001, *p* = 0.0032, and *p* = 0.0239, respectively). In conclusion, our results suggest that the distribution of lymphocyte subsets and expression of PD-1 on T cells before treatment may help predict the outcome of anti-PD-1 treatment in NSCLC patients. In addition, assessing the initial levels of exhausted T cells as well as their decrease upon treatment may also predict response and clinical outcome.

## Introduction

The programmed cell death protein-1 (PD-1) is an immune checkpoint receptor highly expressed on the surface of functionally exhausted T cells after persistent antigen stimulation, in patients with tumors or chronic infections. When engaged by its cognate ligands (PD-L1 and PD-L2), PD-1 mediates a strong inhibitory signal that dampens the T cell effector functions, including direct cytotoxicity and cytokine production. In this setting, targeting the immuno-regulatory axis PD-1/PD-L1 offers a novel approach to restore T cell–mediated antitumor immunity and has become a cornerstone in the current management of several malignancies, including advanced non-small cell lung cancer (NSCLC).

Several antibodies disrupting this axis are currently available in clinical practice for advanced NSCLC. These antibodies, known as immune checkpoint inhibitors (ICI), include the anti-PD-1 monoclonal antibodies nivolumab (1, 2) and pembrolizumab (3, 4), as well as the anti-PD-L1 monoclonal antibody atezolizumab (5). In spite of the impressive results obtained by the use of these agents, a substantial proportion of patients do not experience clinical benefit. For this reason, and because of the high cost of these agents, identifying the most appropriate candidates for PD-1/PD-L1 blockade among patients with NSCLC is a high priority. However, the identification of suitable and affordable biomarkers able to predict either clinical response or resistance has remained elusive. To date, expression of PD-L1 in cancer tissue represents the most deeply investigated marker (6), although several trials showed some inconsistencies regarding its value as predictor of outcomes (7).

For this reason, a significant amount of research has been devoted to identify additional immune system biomarkers, which may provide additional insights into the ways anti-immune checkpoint antibodies exert their effects (8–13). Tumor-infiltrating lymphocytes (TILs) have been shown to represent a key element influencing the behavior of human tumors (14), and the relative abundance and phenotype of specific subsets of TILs have been extensively investigated as potential biomarkers for ICIs (15–17). However, this approach is not feasible for many advanced lung cancer patients because of the limited availability of tumor tissues.

To overcome the need for tissue samples, efforts have been directed on readily accessible samples such as peripheral blood. Various studies have shown that tumor neoantigen-specific T cell clonotypes can be isolated from peripheral blood of cancer patients (18–20). Recent studies have further suggested that sampling peripheral blood may also provide insights into the ongoing immune responses induced by ICI (10, 21).

The discovery of biomarkers able to predict response to checkpoint blocking therapies has now become a priority, although none of these biomarkers have been so far validated as predictors of responsiveness useful in patient selection. In this study, we performed a high-dimensional flow cytometry analysis to investigate the distribution of different lymphocyte subsets in the peripheral blood of NSCLC patients prior and during anti-PD-1 immunotherapy, with the aim of verifying whether specific immune cell signatures could be related to the clinical outcomes.

## Materials and Methods

### Study Population and Assessments of Clinical Outcomes

The study was designed as part of a mono-institutional translational research project at the IRCCS Hospital San Martino in Genoa, Italy. The study included patients receiving nivolumab for advanced NSCLC within the global Italian nivolumab Expanded Access Program (EAP), which was designed to allow patients affected by advanced NSCLC to receive nivolumab in the time period between its registration and its availability in Italy as a therapeutic standard (NCT02475382). The main eligibility criteria for treatment with nivolumab included cytological or histological diagnosis of advanced NSCLC, progression after at least one line of platinum-based chemotherapy for advanced disease, Eastern Cooperative Oncology Group Performance Status (ECOG-PS) ≤ 2. The main exclusion criteria included the need for systemic corticosteroid treatment at a dose >10 mg/die of prednisone (or equivalent) and the presence of uncontrolled brain metastases. Determination of PD-L1 expression was not required for the inclusion within EAP. Eligible patients received nivolumab at 3 mg/Kg every 14 days until death or unacceptable toxicity, or up to 96 weeks from the first administration; in patients with progressive disease, treatment continuation was allowed if clinical benefit was perceived. Those patients who were candidate for receiving nivolumab within the Italian EAP at our Institution were asked to participate in an additional translational research study designed to explore potential predictive biomarkers of outcomes with nivolumab. All subjects gave written informed consent in accordance with the Declaration of Helsinki. The study was approved by our Ethical Committee (N° P.R. 191REG2015) and the participation was not mandatory for receiving nivolumab.

### Response Assessment

The enrolled patients underwent disease assessment after every four doses of nivolumab. Response assessment was performed using the Response Evaluation Criteria in Solid Tumors (RECIST) v. 1.1; additionally, since RECIST may underestimate the activity of ICI, an additional response assessment was performed by using the immune-related response criteria (irRC) (22). In case of PD according to either RECIST or irRC criteria, the subsequent CT scan was performed after two cycles instead of four for confirmation of progression. The best overall response (BOR) achieved during the whole treatment with nivolumab was recorded for both RECIST and irRC according to the following categories: complete response (CR), partial response (PR), stable disease (SD), progressive disease (PD); those patients who died before undergoing at least one radiologic response assessment were categorized as early death (ED). For purposes of our analyses, patients were then aggregated in: (i) a controlled disease (CD) group, which comprised all patients with CR, PR, SD, or (ii) a progressive disease group (PD). ED patients were excluded from the PD group, except in one longitudinal analysis reported in Supplementary Figure 2.

Overall survival (OS) time was recorded from the first administration of nivolumab to the date of death or date at last clinical examination. Progression-free survival (PFS) time was recorded from the first administration to the date of progression according to RECIST (RECIST-PFS) or irRC (irRC-PFS) or date at last clinical examination. If a patient died before experiencing disease progression, PFS was set equal to OS time.

### Blood Collection and Isolation of Peripheral Blood Mononuclear Cells

**Figure 1:**
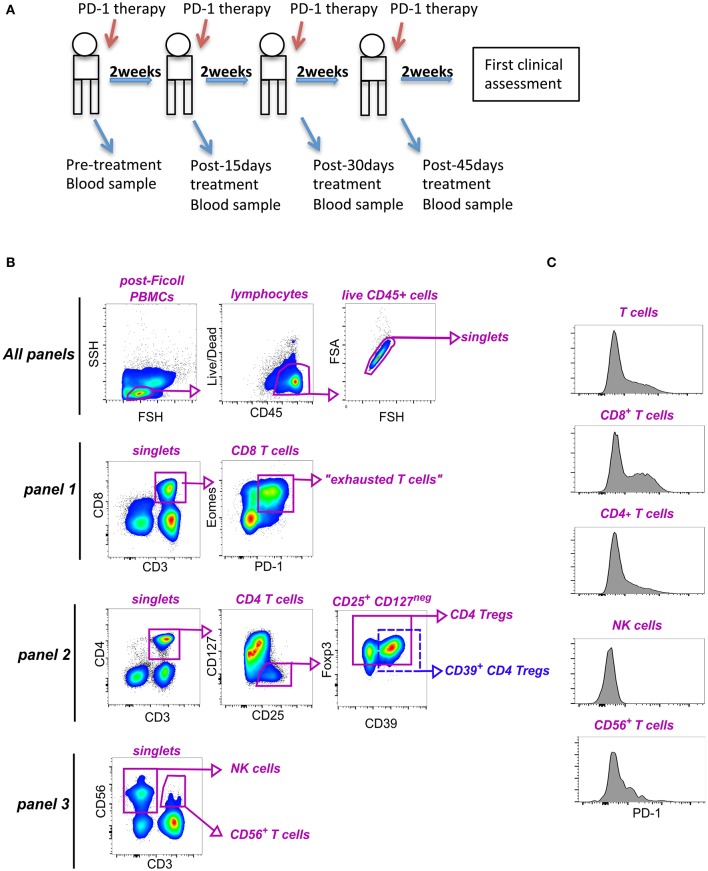
Identification of lymphocyte subpopulations in blood of NSCLC patients. **(A)** Study design. **(B)** Representative gating strategy to identify subsets within PBMCs for immunophenotyping analyses. As shown in the very top row, all data files were first preprocessed to include only CD45^+^ live cells, as well as, singlet events. The remaining gating hierarchy for each of the panels is shown. Panel 1 was used to identify CD8^+^ T cells and exhausted (PD-1^+^ Eomes^+^) T cells (antibodies: CD3, CD8, Eomes, PD-1). Panel 2 allowed characterization of CD4^+^ T cells, as well as total and CD39^+^ Foxp3^+^ Tregs (antibodies CD3, CD4, CD127, CD25, CD39, PD-1, Foxp3). Panel 3 was used to characterize NK cells and CD56^+^ CD3^+^ T cells (antibodies used: CD3, CD56, PD-1). **(C)** Histograms showing expression of PD-1 on CD3^+^ T cells, CD8^+^ T cells, CD4^+^ T cells, CD3^+^ CD56^+^ T cells and NK cells from one representative patient.

In order to evaluate the early landscape of circulating immune cells before and during therapy, blood samples (~20 ml) were collected before each administration of nivolumab, thus, at baseline (Pre-treatment) and at subsequent timings (every 15 days) defined as, post 15D, post 30D, and post 45D (Figure 1A). Upon collection, blood samples were quickly processed by Ficoll-Hypaque (Cedarlane, Canada), density gradient centrifugation to isolate peripheral blood mononuclear cells **(**PBMCs), which were then stored in liquid nitrogen for subsequent flow cytometry analyses. For each sample, white blood cells count (WBCs) was also retrieved and used to calculate absolute number of cells within the different lymphocyte subsets.

### Flow Cytometry

To perform phenotypic analyses, frozen PBMCs were thawed and incubated overnight in RPMI 1640 medium (Lonza, GA, USA) plus 10% FCS and Penicillin /Streptomycin (Euroclone, Italy), at 37°C, as previously described by Gros et al. (19). Cells were then stained with three different multicolor staining panels (mAbs details reported in Supplementary Table 1). For the detection of surface markers, cells were incubated with a mixture of antibodies for 30′, at 4°C. For intranuclear antigens detection, after staining for surface markers, cells were additionally fixed, and permeabilized (Foxp3 Transcription Factor Staining Buffer Set, eBioscience), then incubated with a mixture of antibodies against relevant intranuclear markers (40′ at R.T.). Samples were acquired using Gallios (Beckman Coulter) flow cytometer, and data analyzed using FlowJo 10.3 software (TreeStar Inc.).

### Statistical Analysis

Distributions of all immune biomarker measurements and patients' characteristics (gender, age at enrollment, time since diagnosis, ECOG-PS, number of previous treatments, histotype, disease stage, and smoking habits) were explored and summarized using descriptive statistics. In particular, continuous variables (e.g., biomarkers and age at enrollment) were described through mean, median and range of variation (min-max). Data relative to biomarkers were also dichotomized using median values as cut-off points in order to obtain equally-sized subgroups. All categorical and discrete variables (e.g., gender and ECOG-PS) were expressed in terms of absolute and relative frequencies (percentages). Differences in immune biomarker distribution in sub-groups of patients were assessed using the non-parametric Kruskal-Wallis test. Kaplan-Meier method was applied to describe the effect of each dichotomized immune biomarker on PFS/OS probabilities while the association between all immune biomarkers and relapse/death rates was estimated by means of the Cox regression analysis and expressed as (hazard) ratio (HR), which represents the rate of relapse/death in the higher immune biomarker category relative to the analogous rate in the lower category. Cox regression analysis was adjusted for gender, age at enrollment, time since diagnosis, ECOG-PS, number of previous treatments, and histotype. To evaluate the association between each baseline immune biomarker measurement and RECIST/irRC binary BOR outcome (CD vs. PD), a logistic regression analysis was performed. In this setting, odds ratio (OR), namely the ratio of progressive patients' proportion in the higher immune biomarker category to the analogous proportion in the lower category, was calculated as an index of association, and was adjusted for gender, age at enrollment, time since diagnosis, ECOG-PS, number of previous treatments and histotype. Finally, in order to assess the impact of the RECIST/irRC BOR variable (PD vs. CD) on the time trajectory of all individual immune biomarker measurements from baseline (cycle 1) to post 45D (cycle 4), a random effects regression analysis was applied to log-transformed immune biomarker data. In this context, median ratio (MR), that is the ratio of median immune biomarker value among PD patients and the median immune biomarker value among CD patients, was used as an index of association. In all regression settings, baseline patients' characteristics were taken into consideration as confounding factors and statistical inference on HR/OR/MR was carried out using the likelihood ratio test. Ninety-five percent confidence limits (95% CL) were also computed for all indexes. A two-tailed *p* <0.050 was assumed as statistically significant. Additional details are provided in figure legends. All the analyses were performed using Stata (StataCorp. Stata Statistical Software. Release 13.1. College Station, TX (USA), 2013).

## Results

### Study Population and Patients Outcomes

Globally, 74 patients were enrolled in this study. The baseline clinical and pathological characteristics are reported in Table 1 and were used as covariates in the subsequent correlation analyses.

**Table 1:** Summary of clinical and pathological patients' characteristics.

**Patients' characteristics**	**N**	**%**	**Mean**	**SD**	**Min-Max**
**Gender**					
Male	51	68.9			
Female	23	31.1			
			67.6	9.0	44.0–85.0
**Age**					
≤ 70 years	37	50.0			
> 70 years	37	50.0			
			2.4	2.1	0.0–11.3
**Time since diagnosis**					
≤ 2 years	37	50.0			
>2 years	37	50.0			
***ECOG-PS***					
0	27	36.5			
≥ 1	47	63.5			
**Previous treatments**					
1	30	40.5			
>1	44	58.1			
**Histotype**					
Squamous cell lung cancer	15	20.3			
Adenocarcinoma	59	79.7			
**Smoking habit**					
Non smoker	9	12.2			
Ex-smoker	27	36.5			
Current smoker	38	51.4			
***PD-L1***					
0%	40	54.1			
≥ 1%	7	9.5			
Missing	27	36.5			
Whole sample	74	100.0			

*ECOG-PS, Eastern Cooperative Oncology Group Performance Status*.

Notably, only two patients had EGFR mutation, one harboring exon 19 deletion and one harboring exon 19 deletion and exon 20 insertion, while no ALK or ROS1 rearrangements were reported. The median number of nivolumab administrations was 6 (range: 1–46). Three patients were not evaluable for BOR assessment as the best response CT scan was not available, while one patient was not evaluable for PFS assessment, as he/she did not undergo further CT scans. One patient was considered evaluable for irRC BOR and PFS but not for RECIST BOR and PFS, due to differences between the criteria. All the patients were evaluable for OS analysis. The median OS, RECIST-PFS and irRC-PFS time were 8.60, 1.87, and 1.93 months, respectively. The RECIST-BOR was reported as follows: PR = 10; SD = 14; PD = 30; the irRC-BOR was reported as follows: PR = 9; SD = 19; PD = 27. No complete responses were observed. Overall, 16 patients died before undergoing the first response assessment, and were defined as ED. Blood specimens for PBMC isolation and immunophenotyping were available for 73 patients at baseline, while fewer samples were available at the subsequent time-points.

### Association Between Survival and PB Lymphocyte Parameters at Baseline

To gain insights in the mechanisms underlying the clinical responses to anti-PD-1 therapy in NSCLC patients, we comprehensively evaluated the frequency and phenotype of lymphocyte subsets potentially involved in the response to human tumors upon anti-PD-1 therapy (namely T cells and NK cells) in PBMCs of patients receiving nivolumab. By the use of multicolor flow cytometry (Figures 1B,C), we assessed the frequency of total circulating T cells and NK cells, as well as the relative amount of CD8^+^ and CD4^+^ T cells. Among CD8^+^ T cells, we also quantified the frequency of *bona fide* “exhausted” T cells by gating on PD-1^+^ Eomes^+^ CD8^+^ T cells, as previously described Twyman-Saint Victor et al. (10). In addition, because of their key role in the modulation of immune responses, we investigated the impact of regulatory CD4^+^ CD25^+^ CD127^neg^ Foxp3^+^ T cells (Tregs), including those co-expressing CD39 (CD39^+^ Tregs) (23), in our samples. Given the cytotoxic potential of CD3^+^ CD56^+^ T cells (24), we also identified and monitored this subset in our cohort of patients.

**Figure 2:**
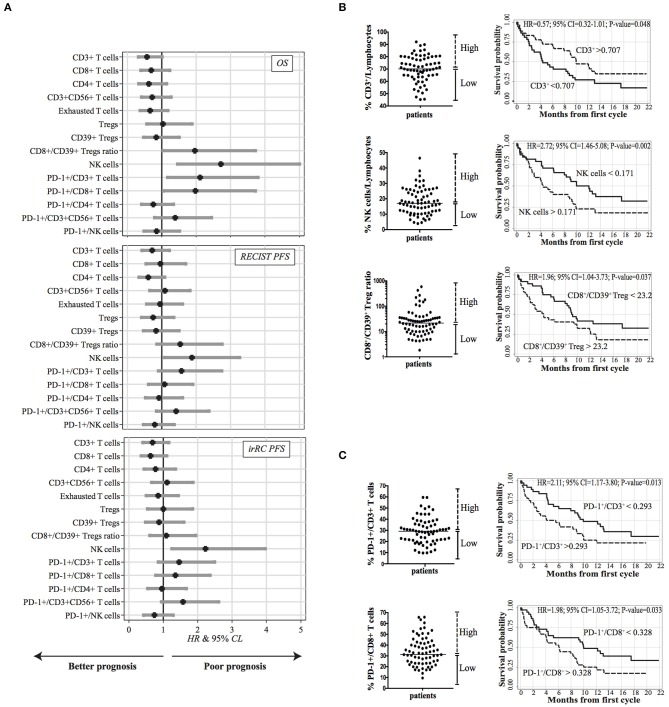
Correlation between baseline PB lymphocyte subsets and survival (*n* = 73). **(A)** Caterpillar plots showing the impact of each immune biomarker evaluated at baseline on overall survival (OS), RECIST, and irRC PFS. HR and corresponding 95% CL were derived from a Cox regression analysis adjusted for gender, age at enrollment, time since diagnosis, ECOG-PS, number of previous treatments and histotype. Vertical line at HR = 1 divides HR associated with a better prognosis (left side) from those associated with a worsen prognosis (right side). **(B)** Kaplan-Meier survival curves illustrating the prognostic effect on OS of CD3^+^ T cells and CD56^+^ CD3^−^ NK cells frequencies, and CD8^+^/CD39^+^ Treg ratio for patients with higher (dotted lines) and lower (solid lines) values. **(C)** Kaplan–Meier survival curves for patients with high or low expression of PD-1 on CD3^+^ T cells and on CD8^+^ T cells. **(B,C)** The median values used as thresholds for categorizing immune biomarkers are indicated in the scatter plots on the left side of each curve.

First, the impact of the baseline immunological status (pre-treatment) on OS, RECIST-PFS and irRC-PFS upon nivolumab treatment was evaluated. Figure 2A and Supplementary Table 2A summarize the effect of each biomarker on patient's life expectancy through Hazard Ratio (HR) point estimates and corresponding 95% CL, obtained from the Cox regression analysis. When HR > 1, higher immune biomarker levels (i.e., greater than median value) are correlated with higher death/relapse rates.

Our results suggest that higher levels of almost all the effector T cell subsets, namely, CD3^+^, CD8^+^, and CD4^+^ T cells were correlated with longer OS and PFS (HR = 0.57; 95% CL = 0.32–1.01; HR = 0.69; 95% CL = 0.39–1.23; HR = 0.62; 95% CL = 0.33–1.14, respectively). Also a higher percentage of circulating exhausted CD8^+^ T cells was associated with longer OS (HR = 0.66; 95% CL = 0.37-1.18) and PFS (HR = 0.85), albeit not reaching statistical significance (95% CL = 0.50–1.44, by irRC only). The role of CD3^+^ CD56^+^ T cells was not clear: patients displaying percentages of CD3^+^ CD56^+^ T cells higher than the median were associated with longer OS (HR = 0.72; 95% CL = 0.41–1.27) but with shorter PFS (HR = 1.09; 95% CL = 0.65–1.82; HR = 1.11, 95% CL = 0.66–1.86, by RECIST and irRC, respectively). Similarly, with regard to total Treg cells and CD39^+^ Treg cells, we did not find any significant correlation between their amounts and OS or PFS, but only a trend toward higher baseline frequency of CD39^+^ Tregs in patients characterized by longer OS (HR = 0.84, 95% CL = 0.47–1.51) and PFS (0.84 RECIST; 0.88 irRC).

Since high values of CD8^+^ T cells to Treg ratio have been correlated to a higher rate of response to immune checkpoint inhibitors (10), we also assessed whether patients with high (CD8^+^ /CD39^+^ Treg) ratio would show better OS and/or clinical benefit. Surprisingly, we did not find any improvement of survival in patients with higher baseline CD8/CD39^+^ Treg ratio; rather, higher values were associated with lower OS (HR = 1.96, 95% CL = 1.04–3.72) and RECIST-PFS (HR = 1.53, 95% CL = 0.85–2.75). This could be due to a trend toward a higher baseline frequency of CD39^+^ Tregs in patients characterized by longer overall survival.

Next, in order to define whether the survival advantage of a higher frequency of effector T cells could be, on the other hand, related to a peculiar expression of PD-1^+^ cells within each subset, we also analyzed the expression of this immune checkpoint on the various sub-populations previously defined (Figure 1C). In contrast with previous reports (25, 26), higher expression levels of PD-1 on CD3^+^ cells and on CD8^+^ T cells were significantly associated with poor OS and PFS and a similar trend was observed on CD3^+^ CD56^+^ T cells (HR = 1.39, 95% CL = 0.79–2.45, HR = 1.42, 95% CL = 0.84–2.37, HR = 1.57, 95% CL = 0.94–2.6 for OS, PFS RECIST and PFS irRC, respectively) (Figure 2A). On the contrary, no correlation between PD-1 levels on CD4^+^ T cells and survival was detected (OS: HR = 0.75, 95% CL = 0.41–1.34; PFS: HR = 0.92, 95% CL = 0.52–1.60; HR = 0.96, 95% CL = 0.55–1.67, by RECIST and irRC, respectively). Overall, these data suggest that higher baseline levels of PD-1 expression on effector T cells may be associated with limited clinical efficacy of nivolumab treatment in advanced NSCLC.

Concerning the innate immune response, high frequencies of NK cells were correlated with lower OS (HR = 2.72, 95% CL = 1.46–5.08) and PFS (HR = 1.88, 95% CL = 1.08–3.26; HR = 2.22, 95% CL = 1.25–3.96, by RECIST and irRC, respectively). Moreover, we found that a discrete subset of NK cells expressed PD-1 on their surface (range: 0.6–10%). Nonetheless, the role of PD-1 expressed by NK cells was unclear, since we did not find a significant correlation with OS (HR = 0.85, 95% CL = 0.48–1.52), whereas only a slight association with PFS was present (HR = 0.8, 95% CL = 0.46–1.36; HR = 0.74, 95% CL = 0.43–1.29, by RECIST and irRC, respectively) (Figure 2A).

Our data were further confirmed using multivariate analyses (Kaplan-Meier survival curves) (Figure 2B). Although statistical significance was not observed for several of the analyzed markers, relative amounts (“High” vs. “Low” groups according to median value of the marker) of circulating CD3^+^ cells and NK cells could predict patients' overall survival time (*p* = 0.048 and *p* = 0.002, respectively). These data reinforce the notion that the relative abundance of CD3^+^ T cells and NK cells could represent, respectively, positive and negative prognostic markers of survival in our cohort. The CD8/CD39^+^ Treg ratio emerged as a biomarker able to predict the clinical outcome, since higher values (> 23.2) at baseline were associated with shorter OS (*p* = 0.037). We also noticed that baseline PD-1 expression was generally correlated with poor clinical outcomes. In particular, high expression of PD-1 on CD3^+^ cells (> 29.3%) and on CD8^+^ T cells (>32.8%) were both significantly associated with shorter OS (*p* = 0.013 and *p* = 0.033, respectively) (Figure 2C).

We further investigated the absolute numbers of the above mentioned immune cell subsets, in order to confirm their impact on OS. In agreement with data showed in Figures 2B–C, patients characterized by high number of circulating CD3^+^ lymphocytes (>1.015 × 10^∧^6/ml) displayed a significant longer OS (*p* = 0.017). Along the same line, we found high absolute number of CD39^+^Tregs, significantly correlated with longer OS (*p* = 0.040), thus supporting the association between a low CD8/CD39^+^ Treg ratio and better survival rate. On the contrary, although not statistically significant (*p* = 0.449), patients characterized by longer OS showed a trend toward a higher absolute number of NK cells. Finally, high levels of circulating PD-1^+^CD3^+^ and PD-1^+^CD8^+^ T cells displayed no correlation with survival (Supplementary Figure 1).

### Baseline PB Immune Features and Radiological Response

**Figure 3:**
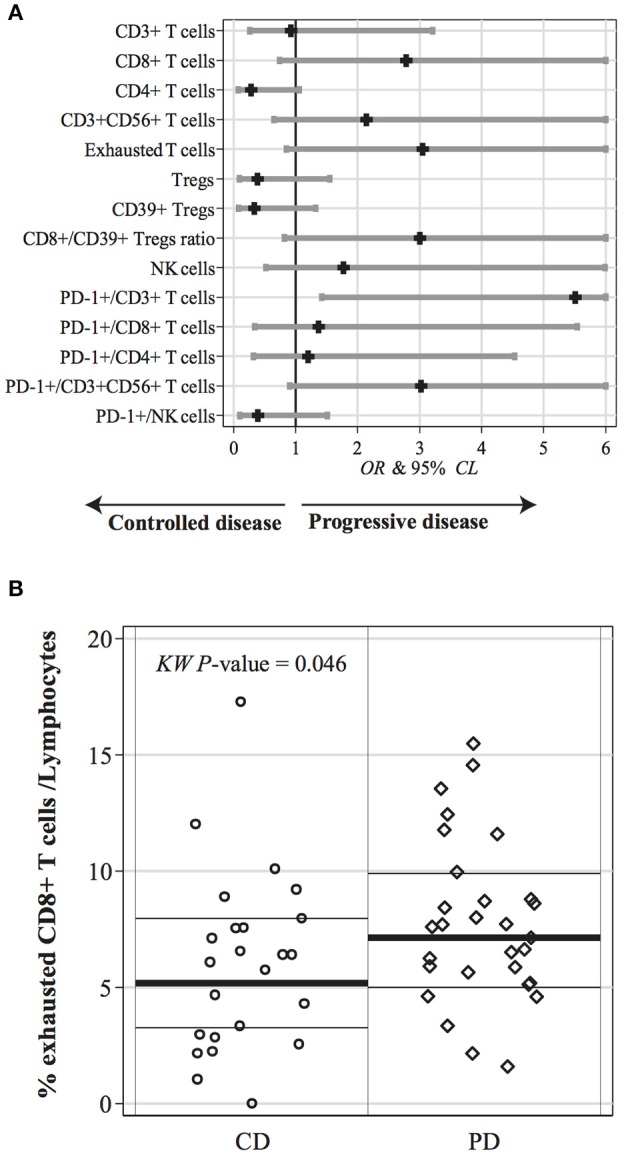
Correlation between PB lymphocyte subsets at baseline and radiological response (*n* = 54). **(A)** Caterpillar plot showing the effect of each immune biomarker evaluated at baseline on the binary (CD vs. PD) best overall response (BOR) according to RECIST criteria. Odds ratio (OR) and corresponding 95% CL, were derived from a logistic regression analysis adjusted for gender, age at enrollment, time since diagnosis, ECOG-PS, number of previous treatments and histotype. Vertical line at OR = 1 divides OR associated with a CD status (left side) from those associated with a PD status (right side). **(B)** Distribution of exhausted CD8^+^ T cell population, at baseline, in CD and PD patients. Statistical comparison was performed using Kruskall-Wallis test (KW P-value). Median values are represented by the thick horizontal line while 25 and 75-th percentiles (inter-quartile difference) are represented by the thin horizontal lines.

Next, we asked whether, beside OS, immunological signatures measured at baseline could also be prognostic of clinical response (i.e., RECIST and irRC). Notably, analyses using irRC criteria did not show any relevant difference from RECIST. In agreement with our data correlating immune biomarkers and OS, higher frequency of total CD3^+^ lymphocytes (HR= 0.92, 95% CI= 0.26–3.21) and total CD4^+^ T cells (HR=0.28, 95% CI= 0.07–1.06, *p* = 0.061) were also associated with controlled disease (Figure 3A). Similarly, higher amounts of both total Tregs and CD39^+^ Tregs correlated with clinical benefit (CD group) (Tregs: HR = 0.38, 95% CI = 0.09–1.55; CD39^+^ Tregs: HR = 0.33, 95% CI = 0.08–1.32). Conversely, a higher frequency of NK cells was associated with a progressive disease (PD) (HR = 1.77, 95% CI = 0.52–5.99). Consistent with previous observation on the negative impact of PD-1 expression on patients' OS, we found that PD patients differed from CD patients by displaying higher level of PD-1 on almost all lymphocyte sub-populations examined (i.e. CD3^+^, CD4^+^, CD8^+^, CD3^+^ CD56^+^ T cells) (Figure 3A and Supplementary Table 2B). In particular, PD-1^+^CD3^+^ T cells were significantly higher in PD patients than in CD patients (p = 0.013). These data corroborate the notion that PD-1 expression on circulating T lymphocytes, at baseline, might negatively impact on patient's clinical response and survival. Surprisingly, in contrast to what observed analyzing OS (Figure 2A), we found that higher frequency of CD8^+^ T cells correlated with progressive disease (HR = 2.78, 95% CI = 0.74–10.43).

By repeating the same multivariate Cox regression analysis including also early-death (ED) patients within the PD group (Supplementary Figure 2), we confirmed higher frequencies of CD3^+^ T cells, CD4^+^ T cells, total and CD39^+^ Tregs in CD vs. PD patients. Conversely, higher amount of CD8^+^ T cells, NK cells, CD8^+^/CD39^+^ Treg ratio and higher PD-1 expression on CD3^+^ T cells and CD3^+^ CD56^+^ T cells characterized PD patients.

We further investigated each of the above-mentioned parameters with univariate analysis obtaining similar results. In particular, while not reaching statistical significance on multivariate model, univariate analysis showed that the impact of exhausted CD8^+^ T cells on the response rate was remarkable. Of note, although exhausted T cells were supposed to be the target sub-population of ICI, the baseline frequency was significantly higher in PD patients than in CD patients (Figure 3B, *p* = 0.046).

### Changes in PB Lymphocyte Parameters During Anti-PD-1 Therapy

Finally, we aimed at identifying whether variations in immunological biomarkers, assessed during the treatment, could associate with disease control. To this end, we performed a longitudinal analysis of blood samples obtained at baseline and before each treatment cycle, up to 6 weeks. Notably, while some studies have reported that therapeutic anti–PD-1 administration hampers detection of PD-1 expression on peripheral blood cells by commercially available antibodies (21, 27, 28), we did not notice any technical issue on this regard. ED patients were excluded from longitudinal analyses, as they could not undergo all the four pre-planned blood sample collections. Thus, blood samples at pre-treatment, post 15D, post 30D, and post 45D were available for 54 patients. No difference was found in longitudinal immune profiles when patients were grouped on the basis of histology or smoking habits (Supplementary Figure 3).

**Figure 4:**
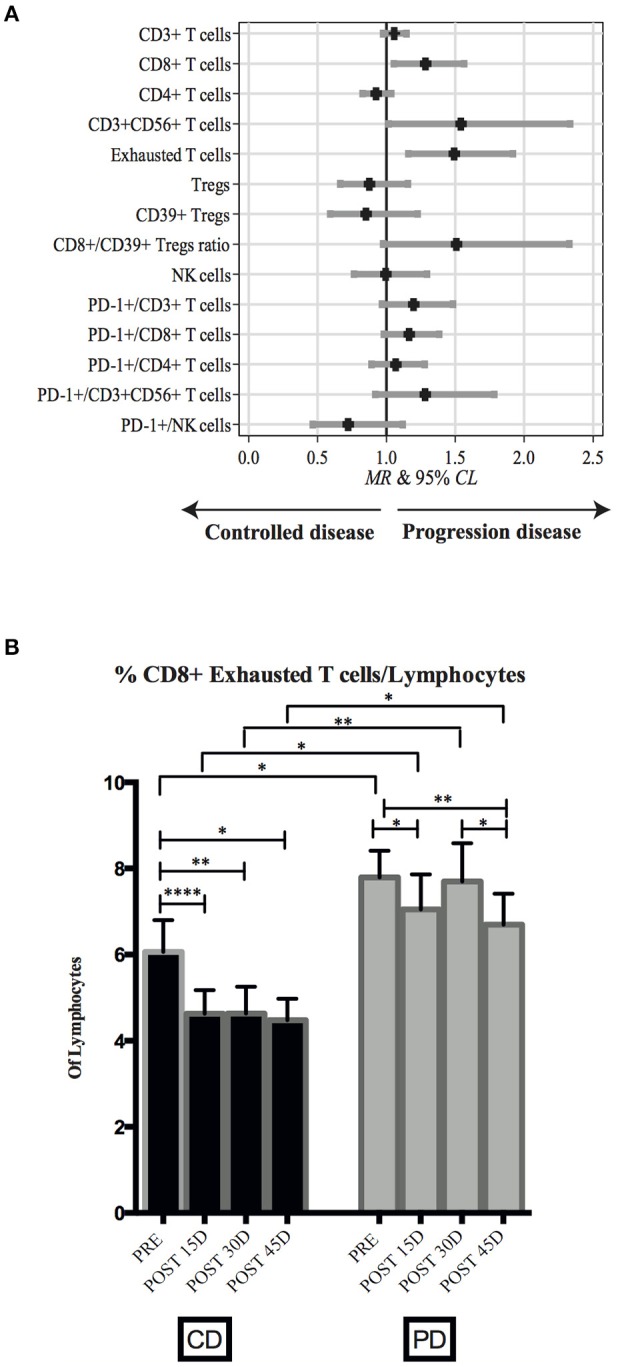
Changes in PB lymphocyte subsets during nivolumab treatment. **(A)** Caterpillar plot showing the relationship between CD vs. PD patients on the longitudinal profile of all immune biomarkers. Median ratio (MR) and corresponding 95% CL were derived from a random effects regression analysis adjusted for gender, age at enrollment, time since diagnosis, ECOG-PS, number of previous treatments, and histotype. Vertical line at MR = 1 divides MR associated with a controlled disease (left side) from those associated with a progressive disease (right side). **(B)** Frequency of exhausted CD8^+^ T cells assessed at four different time points, Pre-treatment (*n* = 54), post 15D (*n* = 48), post 30D (*n* = 45), and post 45D (*n* = 48) in CD and PD patients. Bars represent mean values ±SD. Mann-Whitney test (statistical comparisons between CD and PD groups) or paired non-parametric Wilcoxon test (statistical comparisons within each group) was used for statistical analyses. *p < 0.05, **p < 0.01, ****p < 0.0001.

We found that in PD patients the frequencies of CD8^+^ T cells, exhausted T cells, and CD3^+^ CD56^+^ T cells were significantly higher than median values of the CD group. Figure 4A and Supplementary Table 2C show the relationship between CD vs. PD (BOR) on the longitudinal profile of all immune biomarkers. MR represents the ratio of each immune marker median value in PD–CD groups. These data, again, highlighted the putative role of exhausted T cells as a prognostic biomarker.

Thus, when longitudinally analyzed in each group of patients, we found that the frequency of exhausted T cells among total lymphocytes was lower in CD than PD patients. Interestingly, in CD patients the level of exhausted T cells immediately decreased from baseline after the first cycle of therapy (*p* = < 0.0001), then reaching plateau only at day 45 of treatment (Figure 4B). Conversely, it is worth noting that levels of exhausted T cells in PD patients did not show significant variations across the different time points. Moreover, exhausted T cell levels were consistently higher in PD than in CD patients during treatment.

Similarly, the difference of CD8^+^ T cells and of CD3^+^ CD56^+^ T cells frequencies between CD and PD patients persisted during therapy (*p* = 0.016 and 0.045, respectively, data not shown).

## Discussion

While immunotherapy with PD-1/PD-L1 blocking agents has achieved impressive results in the management of advanced NSCLC, reliable biomarkers of efficacy are still limited. This study was designed to determine whether the proportion of circulating T cell and NK cell sub-populations and the expression of informative cell surface antigens could represent potential predictors of outcomes for NSCLC patients receiving nivolumab. With regards to baseline values, our data show that patients displaying higher frequencies of total CD3^+^ T cells and of distinct T cell subsets (i.e., CD4^+^, CD8^+^ and CD3^+^ CD56^+^ T cells, with the exception of Tregs) achieved a survival advantage upon treatment with nivolumab. Conversely, relative high proportions of NK cells, as well as a higher CD8^+^ /CD39^+^ Treg ratio, were associated with shorter OS and PFS; along this line, also the expression of high levels of PD-1 appeared to play a role in limiting patients' survival. These data are in line with what observed by Mazzaschi and coworkers reporting that in NSCLC a low expression of PD-1 among CD8^+^ TILs was associated with prolonged PFS during treatment with nivolumab (16). In agreement with what found by analyzing OS, our data show that baseline higher frequencies of total CD3^+^ T cells, CD4^+^ T cells, total Tregs and CD39^+^ Tregs were correlated with CD, whereas higher proportions of NK cells, as well as a high CD8^+^ /CD39^+^ Treg ratio, were mostly found in PD patients. A remarkable exception regards the frequency of CD8^+^ T cells that, in contrast to what observed by analyzing patients survival, was significantly associated to PD.

These findings suggest that while some sub-populations associated with survival were also associated with radiological response in a consistent fashion (i.e. CD3^+^ and CD4^+^ effector T cells were associated with improved response and survival, while higher frequencies of NK cells were associated with worse response and survival), other sub-populations appeared to behave differently in terms of response and survival. This apparent inconsistency may be explained by the peculiar mechanism of action of immune checkpoint inhibitors, which might achieve prolonged survival while their effect in terms of response might be underestimated by radiological response evaluation criteria (this is especially true with regards to RECIST).

Despite our results that correlate higher frequencies of NK cells to shorter OS seem relevant, parallel evaluation of absolute numbers of circulating NK cells also suggested their positive association with longer OS. This data is in line with what already documented by Mazzaschi et al. (28), thus suggesting that the specific contribution of NK cells to the response to nivolumab is an aspect that requires further investigations.

Another unexpected result emerging from our analysis is the association of a higher CD8/CD39^+^ Treg ratio with both lack of response and shorter OS. This observation would prompt further investigation at the tumor site, in order to evaluate the relative amount of CD8^+^ T cells and Tregs within neoplastic lesions. However, the current inability to access matched FFPE tissue samples limits our speculation. Nonetheless, in our cohort, the low CD8/CD39^+^ Treg ratio is paralleled by a significant higher level of circulating CD39^+^ Tregs in patients displaying longer OS.

In humans, CD39 characterizes a subset of highly suppressive Tregs. CD39-expressing Tregs are indeed distinguished from their CD39 negative counterparts for higher proliferative, survival and suppressive capacities (29, 30). Notably, a significant expansion of CD39^high^ PD-1^high^ CTLA4hi Foxp3^high^ Tregs has been detected in early lung cancers, thus suggesting that the high level of circulating CD39^+^ Tregs recovered from blood could mirror the high level of CD39^+^ Tregs at the tumor site (31). Functionally, it has been described that intratumoral CD39^+^Treg cells act within tumor-associated tertiary lymphoid structures (TA-TLS) by suppressing antitumor T cell responses (32). Importantly, TA-TLS are frequently described in human lung cancers and correlate with higher frequency of T cells and presence of clonally expanded CD8^+^ PD-1^+^ T cells. Given that intratumoral PD-1^+^ Treg cells express high levels of PD-1, which acts as a stimulatory receptor rather than inhibitory (33), agents blocking PD-1 activity may also target Treg suppressive function within TA-TLS, thus unleashing a powerful immune-mediated response at the tumor site. Thus, it would be interesting to further investigate the expression of immune checkpoints (including PD-1 and CTLA-4) on CD39^+^ Tregs present in our patients.

Interestingly, in line with data regarding the inverse correlation between overall PD-1 expression and OS, we found that at baseline higher PD-1 levels on T cells were strictly associated with poor outcome. This data suggests that baseline PD-1 levels of expression on lymphocytes may represent a relevant predictive biomarker correlated with both survival and response.

Remarkably, univariate analysis showed that the frequency of exhausted T cells at baseline is higher in PD patients, thus highlighting the possible role of this CD8^+^ T cell sub-population as a predictive factor associated with response.

Since our present analysis was performed on PB lymphocytes, our results might not completely mirror what found analyzing CD8^+^ T cells present at the tumor site. Indeed, data collected from metastatic melanoma samples suggest that a higher proportion of exhausted CD8^+^ among TILs predicts response to PD-1/PD-L1 blockade, implying that the relative composition of TIL populations might impact on the outcomes achieved by immune checkpoint inhibitors (17).

Finally, our present data suggest that levels of circulating exhausted T cells during nivolumab therapy may be associated with clinical outcome, though this association needs to be confirmed by larger studies.

Longitudinal peripheral blood samples from patients revealed that, both at baseline and before each treatment cycle, the frequency of exhausted T cells was higher in patients with uncontrolled disease as compared to patients controlling disease. Moreover, in CD group the amount of exhausted T cells declined soon after the first therapy cycle and then remained stable until the fourth administration, whereas in PD patients exhausted T cells levels alternatively increased and decreased at different time points.

These results support the notion that the levels of circulating exhausted T cells may be used to identify patients who will experience clinical disease control upon nivolumab administration. However, additional studies with detailed analysis of levels of exhausted T cell population in peripheral blood during late weeks after PD-1 blockade are needed.

Thus, if these results will be confirmed and extended also at later time points, peripheral blood analysis of exhausted T cell subsets may provide a valuable strategy to predict responses to PD-1–targeted therapy that may assist in the management of lung cancer patients.

We are aware that the possible conclusions of our study might be limited by the relatively small number of evaluated patients, and by the absence of a validation cohort for confirmation of these results in a larger and independent cohort of NSCLC patients. This holds true especially when the longitudinal assessment is taken into account, as some patients did not undergo all the pre-planned sample collections due to early treatment discontinuation. Since we explored a relatively large variety of circulating lymphocyte sub-populations compared to our number of patients, we did not proceed with sub-group analyses based on histology and smoking status, taking also into account the relative disproportion favoring non-squamous histology and smoking history in our population. However, we acknowledge that histology and smoking status might influence tissue and circulating proportions of lymphocytes; hence, further research with a wider patient population and pre-planned end-points based on these parameters are advised. With regards to *EGFR* mutations, while it is known that single-agent immunotherapy has a limited role for patients harboring such mutations (34), our proportion of *EGFR*-mutant patients was too small (two patients harboring different mutations) to draw any relevant conclusion. With regards to the individual responses, the patient with exon 19 deletion experienced disease progression as best response at the first disease assessment with an OS equal to 5.2 months, while the patient harboring exon 19 deletion and exon 20 insertion experienced initial disease control, with a RECIST PFS equal to 2.17 months and an OS equal to 5.6 months. Notably, both patients were heavily pre-treated before receiving immunotherapy (6 and 3 previous lines, respectively), further limiting the possible conclusions on EGFR-mutant NSCLC.

Furthermore, while tissue biomarkers such as immunoscore or the expression of PD-L1 are employed in clinical practice, we were not able to proceed with a comparison between our findings and PD-L1 or immunoscore on tumor samples; the main reason for this limitation relies on the circumstance that, at the time of patient enrollment, the role of PD-L1 was still not consolidated, and tissue collection was not mandatory for the inclusion in the study (thus resulting in many enrolled patients with archival tissue which was not adequate for PD-L1 and immunoscore analysis). Additional studies exploring the role of PD-L1 and immunoscore on circulating lymphocyte sub-populations, as well as other factors which might potentially influence such sub-populations (e.g., circulating auto-antibodies) might be useful for understanding the mechanisms driving the distribution of circulating immune cells.

Finally, it should also be considered that novel immunotherapy-associated biomarkers are emerging in pulmonary oncology; while the determination of tumor mutational burden (TMB) both in tissue and in peripheral blood has achieved mixed results and still needs further evaluations before being considered in clinical practice, the negative role of STK11/LKB1 seems significantly more robust (35, 36). When this study started, information involving the clinical role of STK11/LKB1 was not as acknowledged as it is at present date, hence it was not included as a possible covariate. Future studies involving potential predictors of response in circulating blood should also be compared with the emerging role of STK11/LKB1 and other novel tissue-based biomarkers.

In spite of these limitations, our research might provide an insight on the dynamic immunologic mechanisms leading to different efficacy of PD-1 blocking agents and suggests a potential predictive role of circulating immune cell sub-populations during treatment with nivolumab for advanced NSCLC.

## Data Availability Statement

The datasets analyzed during the current study are available from the corresponding authors (GP and PC) on reasonable request.

## Ethics Statement

The study was approved by the Regional Ethical Committee (N° P.R.191REG2015). Written and informed consent was obtained from patients prior to the collection of specimens.

## Author Contributions

PC, GP, MM, GF, and FG contributed to the conception and design of this study. SO, PC, GP, and IC contributed to the development of methodology. CG, ER, GR, FB, MT, and FG contributed to patient enrollment and recovery of clinical data. SO, PC, GP, IC, AA, SC, SB, and IV contributed to the acquisition of data (acquired and managed patients, provided facilities, etc.). SO, VF, PC, and GP contributed to the analysis and interpretation of data (e.g., statistical analysis, biostatistics, computational analysis). SO, CG, GP, PC, MM, LM, and GF contributed to the writing, review, and/or revision of this manuscript. MD contributed to constructing clinical databases). SO, CG, FG, GF, PC, and GP contributed to the study supervision.

### Conflict of Interest

CG received honoraria from Astra Zeneca, Boehringer Ingelheim, Bristol-Myers Squibb, Merck Sharp & Dohme, Roche. ER received honoraria from Astra Zeneca, Boehringer Ingelheim, Bristol-Myers Squibb, Roche. GR received honoraria from AMGEN, Novartis, and Roche. FG received honoraria from AMGEN, Astra Zeneca, Bristol-Myers Squibb, Boehringer Ingelheim, Celgene, Merck Sharp & Dohme, Pfizer, Pierre Fabre, Roche. The remaining authors declare that the research was conducted in the absence of any commercial or financial relationships that could be construed as a potential conflict of interest.

## Abbreviations

BOR: best overall response
CD: controlled disease
CL: confidence limits
ED: early death
HR: Hazard Ratio
ICI: Immune Checkpoint Inhibitors
irRC: immune-related response criteria
NSCLC: non-small cell lung cancer
OS: overall survival
PBMC: peripheral blood mononuclear cells
PD: progressive disease
PD-1: programmed cell death protein-1
PD-L1: Programmed cell death 1 ligand 1
PFS: Progression-free survival
PR: partial response
RECIST: Response Evaluation Criteria in Solid Tumors
SD: stable disease
TIL: Tumor-Infiltrating Lymphocytes.

## References

[1] Brahmer J Reckamp KL Baas P Crino L Eberhardt WE Poddubskaya E Nivolumab versus docetaxel in advanced squamous-cell non-small-cell lung cancer. N Engl J Med. (2015) 373:123–35. 10.1056/NEJMoa1504627 26028407 PMC4681400

[2] Borghaei H Paz-Ares L Horn L Spigel DR Steins M Ready NE Nivolumab versus docetaxel in advanced nonsquamous non-small-cell lung cancer. N Engl J Med. (2015) 373:1627–39. 10.1056/NEJMoa1507643 26412456 PMC5705936

[3] Herbst RS Baas P Kim DW Felip E Perez-Gracia JL Han JY Pembrolizumab versus docetaxel for previously treated, PD-L1-positive, advanced non-small-cell lung cancer (KEYNOTE-010): a randomised controlled trial. Lancet. (2016) 387:1540–50. 10.1016/S0140-6736(15)01281-7 26712084

[4] Reck M Rodriguez-Abreu D Robinson AG Hui R Csoszi T Fulop A. Pembrolizumab versus chemotherapy for PD-L1-positive non-small-cell lung cancer. N Engl J Med. (2016) 375:1823–33. 10.1056/NEJMoa1606774 27718847

[5] Rittmeyer A Barlesi F Waterkamp D Park K Ciardiello F von Pawel J. Atezolizumab versus docetaxel in patients with previously treated non-small-cell lung cancer (OAK): a phase 3, open-label, multicentre randomised controlled trial. Lancet. (2017) 389:255–65. 10.1016/S0140-6736(16)32517-X 27979383 PMC6886121

[6] Munari E Zamboni G Lunardi G Marchionni L Marconi M Sommaggio M. PD-L1 Expression heterogeneity in non-small cell lung cancer: defining criteria for harmonization between biopsy specimens and whole sections. J Thorac Oncol. (2018) 13:1113–20. 10.1016/j.jtho.2018.04.017 29704674

[7] Lisberg A Garon EB. The value of PD-L1 testing in non-small-cell lung cancer. JAMA Oncol. (2016) 2:571–2. 10.1001/jamaoncol.2016.0043 26986923

[8] Herbst RS Soria JC Kowanetz M Fine GD Hamid O Gordon MS. Predictive correlates of response to the anti-PD-L1 antibody MPDL3280A in cancer patients. Nature. (2014) 515:563–7. 10.1038/nature14011 25428504 PMC4836193

[9] Tumeh PC Harview CL Yearley JH Shintaku IP Taylor EJ Robert L. PD-1 blockade induces responses by inhibiting adaptive immune resistance. Nature. (2014) 515:568–71. 10.1038/nature13954 25428505 PMC4246418

[10] Twyman-Saint Victor C Rech AJ Maity A Rengan R Pauken KE Stelekati E. Radiation and dual checkpoint blockade activate non-redundant immune mechanisms in cancer. Nature. (2015) 520:373–7. 10.1038/nature14292 25754329 PMC4401634

[11] Dronca RS Liu X Harrington SM Chen L Cao S Kottschade LA. T cell Bim levels reflect responses to anti-PD-1 cancer therapy. JCI Insight. (2016) 1:e86014. 10.1172/jci.insight.86014 27182556 PMC4863706

[12] Hannani D Vetizou M Enot D Rusakiewicz S Chaput N Klatzmann D Anticancer immunotherapy by CTLA-4 blockade: obligatory contribution of IL-2 receptors and negative prognostic impact of soluble CD25. Cell Res. (2015) 25:208–24. 10.1038/cr.2015.3 25582080 PMC4650573

[13] Powles T Eder JP Fine GD Braiteh FS Loriot Y Cruz C. MPDL3280A (anti-PD-L1) treatment leads to clinical activity in metastatic bladder cancer. Nature. (2014) 515:558–62. 10.1038/nature13904 25428503

[14] Galon J Costes A Sanchez-Cabo F Kirilovsky A Mlecnik B Lagorce-Pages C. Type, density, and location of immune cells within human colorectal tumors predict clinical outcome. Science. (2006) 313:1960–4. 10.1126/science.1129139 17008531

[15] Uryvaev A Passhak M Hershkovits D Sabo E Bar-Sela G The role of tumor-infiltrating lymphocytes (TILs) as a predictive biomarker of response to anti-PD1 therapy in patients with metastatic non-small cell lung cancer or metastatic melanoma. Med Oncol. (2018) 35:25 10.1007/s12032-018-1080-0 29388007

[16] Mazzaschi G Madeddu D Falco A Bocchialini G Goldoni M Sogni F. Low PD-1 expression in cytotoxic CD8(+) tumor-infiltrating lymphocytes confers an immune-privileged tissue microenvironment in NSCLC with a prognostic and predictive value. Clin Cancer Res. (2018) 24:407–19. 10.1158/1078-0432.CCR-17-2156 29074606

[17] Daud AI Loo K Pauli ML Sanchez-Rodriguez R Sandoval PM Taravati K. Tumor immune profiling predicts response to anti-PD-1 therapy in human melanoma. J Clin Invest. (2016) 126:3447–52. 10.1172/JCI87324 27525433 PMC5004965

[18] Rizvi NA Hellmann MD Snyder A Kvistborg P Makarov V Havel JJ. Cancer immunology. Mutational landscape determines sensitivity to PD-1 blockade in non-small cell lung cancer. Science. (2015) 348:124–8. 10.1126/science.aaa1348 25765070 PMC4993154

[19] Gros A Parkhurst MR Tran E Pasetto A Robbins PF Ilyas S. Prospective identification of neoantigen-specific lymphocytes in the peripheral blood of melanoma patients. Nat Med. (2016) 22:433–8. 10.1038/nm.4051 26901407 PMC7446107

[20] van Rooij N van Buuren MM Philips D Velds A Toebes M Heemskerk B. Tumor exome analysis reveals neoantigen-specific T-cell reactivity in an ipilimumab-responsive melanoma. J Clin Oncol. (2013) 31:e439–42. 10.1200/JCO.2012.47.7521 24043743 PMC3836220

[21] Kamphorst AO Pillai RN Yang S Nasti TH Akondy RS Wieland A. Proliferation of PD-1+ CD8 T cells in peripheral blood after PD-1-targeted therapy in lung cancer patients. Proc Natl Acad Sci USA. (2017) 114:4993–8. 10.1073/pnas.1705327114 28446615 PMC5441721

[22] Kim HK Heo MH Lee HS Sun JM Lee SH Ahn JS. Comparison of RECIST to immune-related response criteria in patients with non-small cell lung cancer treated with immune-checkpoint inhibitors. Cancer Chemother Pharmacol. (2017) 80:591–8. 10.1007/s00280-017-3396-4 28733892

[23] Borsellino G Kleinewietfeld M Di Mitri D Sternjak A Diamantini A Giometto R. Expression of ectonucleotidase CD39 by Foxp3+ Treg cells: hydrolysis of extracellular ATP and immune suppression. Blood. (2007) 110:1225–32. 10.1182/blood-2006-12-064527 17449799

[24] Pittet MJ Speiser DE Valmori D Cerottini JC Romero P. Cutting edge: cytolytic effector function in human circulating CD8+ T cells closely correlates with CD56 surface expression. J Immunol. (2000) 164:1148–52. 10.4049/jimmunol.164.3.1148 10640724

[25] Pauken KE Wherry EJ. Overcoming T cell exhaustion in infection and cancer. Trends Immunol. (2015) 36:265–76. 10.1016/j.it.2015.02.008 25797516 PMC4393798

[26] Chen L Han X. Anti-PD-1/PD-L1 therapy of human cancer: past, present, and future. J Clin Invest. (2015) 125:3384–91. 10.1172/JCI80011 26325035 PMC4588282

[27] Topalian SL Hodi FS Brahmer JR Gettinger SN Smith DC McDermott DF. Safety, activity, and immune correlates of anti-PD-1 antibody in cancer. N Engl J Med. (2012) 366:2443–54. 10.1056/NEJMoa1200690 22658127 PMC3544539

[28] Mazzaschi G Facchinetti F Missale G Canetti D Madeddu D Zecca A. The circulating pool of functionally competent NK and CD8+ cells predicts the outcome of anti-PD1 treatment in advanced NSCLC. Lung Cancer. (2019) 127:153–63. 10.1016/j.lungcan.2018.11.038 30642544

[29] Gu J Ni X Pan X Lu H Lu Y Zhao J Human CD39(hi) regulatory T cells present stronger stability and function under inflammatory conditions. Cell Mol Immunol. (2017) 14:521–8. 10.1038/cmi.2016.30 27374793 PMC5518817

[30] Lu Y Wang X Gu J Lu H Zhang F Li X. iTreg induced from CD39(+) naive T cells demonstrate enhanced proliferate and suppressive ability. Int Immunopharmacol. (2015) 28:925–30. 10.1016/j.intimp.2015.03.039 25864618

[31] Lavin Y Kobayashi S Leader A Amir ED Elefant N Bigenwald C. Innate immune landscape in early lung adenocarcinoma by paired single-cell analyses. Cell. (2017) 169:750–65.e17. 10.1016/j.cell.2017.04.014 28475900 PMC5737939

[32] Joshi NS Akama-Garren EH Lu Y Lee DY Chang GP Li A. Regulatory T cells in tumor-associated tertiary lymphoid structures suppress anti-tumor T cell responses. Immunity. (2015) 43:579–90. 10.1016/j.immuni.2015.08.006 26341400 PMC4826619

[33] Whiteside TL Demaria S Rodriguez-Ruiz ME Zarour HM Melero I. Emerging opportunities and challenges in cancer immunotherapy. Clin Cancer Res. (2016) 22:1845–55. 10.1158/1078-0432.CCR-16-0049 27084738 PMC4943317

[34] Santaniello A Napolitano F Servetto A De Placido P Silvestris N Bianco C. Tumour microenvironment and immune evasion in EGFR addicted NSCLC: hurdles and possibilities. Cancers. (2019) 11:1419. 10.3390/cancers11101419 31554160 PMC6826622

[35] Teng F Meng X Kong L Yu J. Progress and challenges of predictive biomarkers of anti PD-1/PD-L1 immunotherapy: A systematic review. Cancer Lett. (2018) 414:166–73. 10.1016/j.canlet.2017.11.014 29155348

[36] Arora S Velichinskii R Lesh RW Ali U Kubiak M Bansal P. Existing and emerging biomarkers for immune checkpoint immunotherapy in solid tumors. Adv Ther. (2019) 36:2638. 10.1007/s12325-019-01051-z 31410780 PMC6778545

