# Supplementary material for: Association Between Response to Nivolumab Treatment and Peripheral Blood Lymphocyte Subsets in Patients With Non-small Cell Lung Cancer
Source: Front Immunol. 2020 Feb 7;11:125. doi: 10.3389/fimmu.2020.00125 (PMC7018763; doi:10.3389/fimmu.2020.00125)
Supplement: Supplementary file 1 [file Data_Sheet_1.docx]

**SUPPLEMENTARY MATERIAL**

**Supplementary Figures**

**Supplementary Figure 1**.

Kaplan-Meier survival curves illustrating the prognostic effect on OS of baseline CD3^+^ T cells, CD56^+^ CD3^~~-~~^ NK cells and CD39^+^ Treg cells for patients with higher (dotted lines) and lower (solid lines) absolute cell counts **(left column).** Kaplan–Meier survival curves for patients with high or low absolute numbers of PD-1^+^ CD3^+^ T cells and of PD-1^+^ CD8^+^ T cells (**right column).** The median values used as cut off are showed in the graphs. Values refer to absolute count (10^6/ml).

**Supplementary Figure 2**

Correlation between baseline immune biomarkers and best overall response (BOR).

Caterpillar plot showing the effect of each biomarker evaluated at baseline on PD+ ED vs CD, BOR according to RECIST criteria. OR, represented by a plus sign, and corresponding 95% confidence limits (95%CL, represented by horizontal “whiskers”) were derived from a logistic regression analysis adjusted for gender, age at enrollment, time since diagnosis, ECOG-PS, number of previous treatments and histotype. OR is to be interpreted as the ratio of the proportion of progressive patients in higher immune biomarker levels to the analogous proportion in lower levels. Vertical line at OR = 1 divides OR associated with a responsive disease (left side) from those associated with a progressive disease (right side). Statistically significant P-values derived from the likelihood ratio test are reported in the graph.

**A**

**B**

Squamous cell lung cancer

Adenocarcinoma

Non-/Ex- smokers

Current smokers

**Supplementary Figure 3**

**(A)** Caterpillar plot showing the relationship between histology (Adenocarcinoma vs Squamous cell lung cancer) and the frequencies of the peripheral blood immune subsets. **(B)** Caterpillar plot showing the relationship between patients’ smoking habits (Current vs Non-/Ex-smokers) and the frequencies of the peripheral blood immune subsets. MR (median ratios), represented by a plus sign, and corresponding 95% confidence limits (95%CL) represented by horizontal “whiskers”, were derived from a random effects regression modeling applied to log-transformed immune-marker data. All MR are adjusted for gender, age at enrollment, time since diagnosis, ECOG-PS, number of previous treatments and RECIST-BOR. In panel A, each MR is given by the ratio between the median value of an immune-marker of patients with Adenocarcinoma and the median value of the same immune-marker in patients with Squamous cell lung cancer. In Panel B, each MR represents the same ratio in Current smokers versus Non-/Ex-smokers. Vertical line at MR = 1 divides MR associated with Squamous cell lung cancer histotype (Panel A, left side) or non-/ex-smokers (Panel B, left side) from those associated with adenocarcinoma (Panel A, right side) or current smokers (Panel B, right side).

**Supplementary Table 1.** Antibodies and reagents used for flow cytometry

| **Antigen** | **Fluorochrome** | **Clone** | **Source** | **Dilution** | **Cod. n.** |
| --- | --- | --- | --- | --- | --- |
| **CD127** | PE/ DAZZLE | A019D5 | Biolegend | 1:100 | 351336 |
| **CD25** | BV421 | M-A251 | BD | 1:25 | 562442 |
| **CD3** | APC-R700 | UCHT1 | BD | 1:100 | 565119 |
| **CD3** | VioBlue | BW264/56 | Miltenyi | 1:100 | 130-094-363 |
| **CD39** | BB515 | TU66 | BD | 1:25 | 565469 |
| **CD4** | PC7 | SFCI12T4D11 | Coulter | 1:200 | 737660 |
| **CD45** | APC-H7 | 2D1 | BD | 1:100 | 560178 |
| **CD56** | APC | AF12-7H3 | Miltenyi | 1:200 | 130-090-843 |
| **CD56** | PC7 | N901 (HLDA6) | Coulter | 1:100 | A21692 |
| **CD69** | PE-CF594 | FN50 | BD | 1:50 | 562617 |
| **CD8** | APC | RPA-T8 | BD | 1:100 | 561953 |
| **EOMES** | FITC | WD1928 | eBioscience | 1:50 | 11-4877-42 |
| **FoxP3** | AlexaFluor 647 | 259D/C7 | BD | 1:50 | 560045 |
| **Granzyme B** | PE- CF594 | GB11 | BD | 1:200 | 562462 |
| **Ki-67** | Per-CP-Cy 5.5 | B56 | BD | 1:100 | 561284 |
| **KLRG1** | PE-Cy 7 | 2F1/KLRG1 | Biolegend | 1:75 | 138416 |
| **PD1** | PE | J105 | eBioscience | 1:75 | 12-2799-42 |
| **TIM-3** | BV421 | 7D3 | BD | 1:200 | 565563 |

| **Reagent** | **Source** | **Dilution** | **Cod. n.** |
| --- | --- | --- | --- |
| **7-AAD** | BD | 1:20 | 559925 |
| **Fixable Aqua Dead dye** | Invitrogen | 1:500 | L34957 |

**Supplementary Table 2**

1. HR and corresponding 95% CL were derived from a Cox regression analysis adjusted for gender, age at enrollment, time since diagnosis, ECOG-PS, number of previous treatments and histotype. 95%CL: 95% confidence limits for MR; P-value: probability level associated with the likelihood ratio test.

| Biomarker | HR | Inf | Sup | P-value |
| --- | --- | --- | --- | --- |
| CD3+ T cells | 0.57 | 0.32 | 1.01 | 0.048 |
| CD8+ T cells | 0.69 | 0.39 | 1.23 | 0.208 |
| CD4+ T cells | 0.62 | 0.33 | 1.14 | 0.123 |
| CD3+CD56+ T cells | 0.72 | 0.41 | 1.27 | 0.256 |
| Exhausted T cells | 0.66 | 0.37 | 1.18 | 0.162 |
| Tregs | 1.03 | 0.56 | 1.88 | 0.930 |
| CD39+ Tregs | 0.84 | 0.47 | 1.51 | 0.558 |
| CD8+/CD39+ Tregs ratio | 1.96 | 1.04 | 3.72 | 0.037 |
| NK cells | 2.72 | 1.46 | 5.08 | 0.002 |
| PD-1+/CD3+ T cells | 2.11 | 1.17 | 3.80 | 0.013 |
| PD-1+/CD8+ T cells | 1.98 | 1.05 | 3.72 | 0.033 |
| PD-1+/CD4+ T cells | 0.75 | 0.41 | 1.34 | 0.326 |
| PD-1+/CD3+CD56+ T cells | 1.39 | 0.79 | 2.45 | 0.260 |
| PD-1+/NK cells | 0.85 | 0.48 | 1.52 | 0.592 |

1. Odds ratio (OR) and corresponding 95% CL, were derived from a logistic regression analysis adjusted for gender, age at enrollment, time since diagnosis, ECOG-PS, number of previous treatments and histotype. 95%CL: 95% confidence limits for MR; P-value: probability level associated with the likelihood ratio test.

| Biomarker | OR | Inf | Sup | P-value |
| --- | --- | --- | --- | --- |
| CD3+ T cells | 0.92 | 0.26 | 3.21 | 0.896 |
| CD8+ T cells | 2.78 | 0.74 | 10.43 | 0.128 |
| CD4+ T cells | 0.28 | 0.07 | 1.06 | 0.061 |
| CD3+CD56+ T cells | 2.14 | 0.65 | 7.07 | 0.213 |
| Exhausted T cells | 3.05 | 0.85 | 10.96 | 0.087 |
| Tregs | 0.38 | 0.09 | 1.55 | 0.176 |
| CD39+ Tregs | 0.33 | 0.08 | 1.32 | 0.118 |
| CD8+/CD39+ Tregs ratio | 3.00 | 0.82 | 10.92 | 0.096 |
| NK cells | 1.77 | 0.52 | 5.99 | 0.358 |
| PD-1+/CD3+ T cells | 5.51 | 1.42 | 21.31 | 0.013 |
| PD-1+/CD8+ T cells | 1.37 | 0.34 | 5.54 | 0.662 |
| PD-1+/CD4+ T cells | 1.20 | 0.32 | 4.53 | 0.792 |
| PD-1+/CD3+CD56+ T cells | 3.02 | 0.90 | 10.14 | 0.073 |
| PD-1+/NK cells | 0.39 | 0.10 | 1.51 | 0.174 |

1. MR: median ratio derived from a random effects regression analysis and adjusted for gender, age at enrollment, time since diagnosis, ECOG-PS, number of previous treatments and histotype; 95%CL: 95% confidence limits for MR; P-value: probability level associated with the likelihood ratio test.

| Biomarker | MR | Inf | Sup | P-value |
| --- | --- | --- | --- | --- |
| CD3+ T cells | 1.06 | 0.97 | 1.14 | 0.187 |
| CD8+ T cells | 1.28 | 1.05 | 1.56 | 0.016 |
| CD4+ T cells | 0.92 | 0.82 | 1.03 | 0.172 |
| CD3+CD56+ T cells | 1.54 | 1.01 | 2.33 | 0.045 |
| Exhausted T cells | 1.49 | 1.15 | 1.91 | 0.003 |
| Tregs | 0.88 | 0.66 | 1.15 | 0.353 |
| CD39+ Tregs | 0.85 | 0.59 | 1.22 | 0.392 |
| CD8+/CD39+ Tregs ratio | 1.51 | 0.97 | 2.32 | 0.070 |
| NK cells | 1.00 | 0.76 | 1.29 | 0.969 |
| PD-1+/CD3+ T cells | 1.20 | 0.96 | 1.48 | 0.104 |
| PD-1+/CD8+ T cells | 1.17 | 0.98 | 1.38 | 0.085 |
| PD-1+/CD4+ T cells | 1.07 | 0.89 | 1.27 | 0.483 |
| PD-1+/CD3+CD56+ T cells | 1.28 | 0.91 | 1.78 | 0.150 |
| PD-1+/NK cells | 0.72 | 0.46 | 1.11 | 0.147 |
